# Emergence of Deep Learning in Knee Osteoarthritis Diagnosis

**DOI:** 10.1155/2021/4931437

**Published:** 2021-11-10

**Authors:** Pauline Shan Qing Yeoh, Khin Wee Lai, Siew Li Goh, Khairunnisa Hasikin, Yan Chai Hum, Yee Kai Tee, Samiappan Dhanalakshmi

**Affiliations:** ^1^Department of Biomedical Engineering, Faculty of Engineering, Universiti Malaya, Kuala Lumpur 50603, Malaysia; ^2^Sports Medicine Unit, Faculty of Medicine, Universiti Malaya, Kuala Lumpur 50603, Malaysia; ^3^Department of Mechatronics & Biomedical Engineering, Universiti Tunku Abdul Rahman, Sungai Long 43000, Malaysia; ^4^Department of Electronics and Communication Engineering, SRM Institute of Science and Technology, Kattankulathur, Chengalpattu 603203, India

## Abstract

Osteoarthritis (OA), especially knee OA, is the most common form of arthritis, causing significant disability in patients worldwide. Manual diagnosis, segmentation, and annotations of knee joints remain as the popular method to diagnose OA in clinical practices, although they are tedious and greatly subject to user variation. Therefore, to overcome the limitations of the commonly used method as above, numerous deep learning approaches, especially the convolutional neural network (CNN), have been developed to improve the clinical workflow efficiency. Medical imaging processes, especially those that produce 3-dimensional (3D) images such as MRI, possess ability to reveal hidden structures in a volumetric view. Acknowledging that changes in a knee joint is a 3D complexity, 3D CNN has been employed to analyse the joint problem for a more accurate diagnosis in the recent years. In this review, we provide a broad overview on the current 2D and 3D CNN approaches in the OA research field. We reviewed 74 studies related to classification and segmentation of knee osteoarthritis from the Web of Science database and discussed the various state-of-the-art deep learning approaches proposed. We highlighted the potential and possibility of 3D CNN in the knee osteoarthritis field. We concluded by discussing the possible challenges faced as well as the potential advancements in adopting 3D CNNs in this field.

## 1. Introduction

Osteoarthritis (OA) is one of the most prevalent degenerative musculoskeletal diseases. This disease is affecting almost 5% of the global population [1]. The knee is the most common joint affected by OA, which is characterized by irreversible degeneration of the articular cartilage at the ends of the bones such as femoral, tibial, and patella cartilages [2]. Knee osteoarthritis (knee OA) is a progressive disease that affects the entire knee joint. Knee OA is a condition driven by mechanical wear and tear as well as biochemical changes. Known risk factors for OA include aging, obesity [3], and previous knee injuries [4]. OA causes pain that limits function and reduces one's quality of life. The joint damage in OA is irreversible, and definitive treatment requires total knee replacement (TKR), which is expensive and has a short life span especially for the obese individuals [5]. Therefore, early detection of knee OA is crucial for initiation of therapy such as weight reduction and exercises that has been found to be effective in halting knee OA progression and delaying TKR [3, 6].

Current radiographic grading scales for OA rely primarily on Kellgren–Lawrence grading which examines the changes shown on X-ray plain radiography images. However, this approach causes delay in OA diagnosis because the bony changes only appear in advanced conditions. Besides X-ray, other imaging modalities such as magnetic resonance imaging can utilize several OA soft tissue biomarkers such as cartilage and meniscus degeneration and also deformation of the subchondral and trabecular bone to determine the onset of knee OA [1]. There exist different types of OA-related segmentation or classification models for assessing the knee which are generally classified into classical methods and deep learning (DL) methods [7, 8]. In current clinical practices, evaluation of OA severity is normally performed visually with radiography images, which is prone to interrater variability and time consumption for large datasets [9].

Recent studies have adapted artificial intelligence (AI) and have increasingly recognized the role of deep learning in the medical field, including computer-aided knee OA diagnosis [10, 11] which is aimed to reduce uncertainties in diagnosis due to human error [12]. The significant motivation in the development of AI in OA research is the availability of huge repositories of clinical and imaging data such as through Osteoarthritis Initiative (OAI) [13]. There are different types of architecture of deep learning such as convolutional neural network (CNN), recurrent neural network (RNN), recursive neural network, and unsupervised pretrained network (UPN) [8].

**Figure 1:**
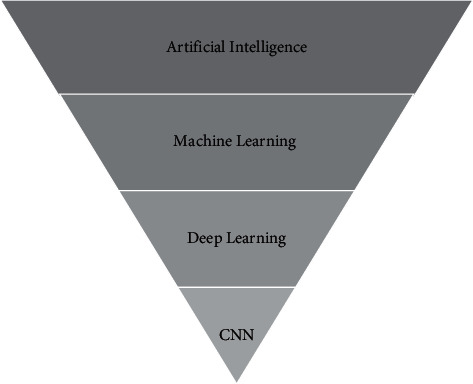
Category of CNN under the umbrella of artificial intelligence.

In the field of medical imaging classification tasks, assessing imaging biomarkers via end-to-end deep neural networks can support the clinicians to provide a more precise diagnosis such as predicting the incident, severity, or progression of a disease or even a clinical outcome. The use of deep learning, especially with convolutional neural networks, is prevalent as it has shown validated results as compared to human practitioners' manual methods or classical methods [8, 12]. Deep learning methods such as CNN learn complex features by extracting visual features automatically using combinations of series of transformations in the model architecture [11, 14].  Figure 1 illustrates the category of CNN under the umbrella of AI. CNN is a form of deep learning which falls under the machine learning category under the umbrella of artificial intelligence (AI). CNNs are robust with low complexity and easy to train where the network learns throughout the optimization process with a reduced number of parameters [15]. The general architecture of CNN involves an input layer, hidden layers associated with a series of image filters with layers of feed-forward networks where image filters are projected onto the input image, and output layer where the feature will be extracted [8, 14].

Clinicians often use a variety of a patient's data to diagnose OA. The data that can be used in medical diagnosis studies are medical image data, usually DICOM images from different modalities such as ultrasound imaging, computed tomography (CT), X-ray, or magnetic resonance imaging (MRI), and nonmedical image data such as statistical data, subject demographic information, and health behaviour information. One of the critical issues in deep learning is overfitting due to the high numbers of parameters and complexity of regularization techniques in the model. Hence, to ensure generalization of the model, the data are typically categorized into three sets: training set for hyperparameter optimization, validation set for overfitting control, and test set [16].

## 2. Nonimaging-Based Deep Learning

Electronic health record datasets contain a diverse clinical dataset of patient information such as diagnosis, treatment plans, test results, and medical history including imaging data which are radiography medical images. Demographic information, personal characteristics, symptoms, lifestyles, and health behaviour of patients, either self-reported or assessed, are variables that are included in patient's assessment. Not only imaging data but also these nonimaging data contain rich sources of important information that might play important roles in disease predictions [4].

The studies on deep learning in OA using nonimage data are limited as most studies focused on imaging-based deep learning models in OA diagnosis. Early diagnosis of OA is challenging as it is a complex disease that might be due to a lot of risk factors such as age, sex, body weight, body mass index (BMI), family history of disease, activities of daily living, or even job scope [2, 4]. Besides demographic information, radiographic risk factors such as KL-grade can be utilized as well. Although radiographic images remain the “gold standard” of OA diagnosis, statistical data including the health behaviour information of the patients will be convenient for knee OA progression prediction. The nonimaging data are easier to obtain, are cost- and time-efficient compared to medical images, and have shown ability in OA diagnosis [4, 9].

Studies have shown that utilizing only nonimaging data can be a promising approach to prescreen prevalence of osteoarthritis [4, 9]. Lim et al. [9] proposed a deep learning model of deep neural network (DNN) with eight hidden layers with scaled principal component analysis (PCA) for early OA diagnosis using demographic and personal information data. An area under the receiver operating characteristics curve (AUC) of 76.8% has been achieved using DNN with scaled PCA in the classification task on predicting the presence and the risk of OA.

Unlike the study in [9] that performed two-class classification where it only takes into account subjects diagnosed with OA and subjects without OA into classification, Christodoulou et al. [4] separated the subjects into 3 classes: incidence, progressive, and nonexposed OA. DNN was also investigated by Christodoulou et al. [4] to classify and detect OA without medical images, solely depending on 141 risk factors dataset. The data included are all self-reported data on health behaviour, including the joint symptoms and disability of the subjects with or without KOA from baseline visit. Different DNN architectures were tested on six subgroups which are based on the gender (male or female), age (below or above 70), and obesity (obese or nonobese) respectively. The authors discussed the possibility of creating more accurate diagnostic models by subgroups especially for patients with obesity. Based on the overall performance, DNN with one hidden layer and 50 modes has the highest classification accuracy of 79.39%.

Several studies have integrated nonimaging data and medical images in the OA detection architecture. Studies have shown that inclusion of nonimaging data such as demographics will improve the DL model's performance to predict OA severity and progression [11, 17, 18], staging lesion severity [19, 20], TKR [21], and even knee pain [22].  Table 1 presents the studies conducted with nonimaging data.

## 3. Imaging-Based Deep Learning

The structural hallmarks of whole joint involvement in this degenerative disease include the joint space narrowing, which is indirectly caused by cartilage and menisci loss, and subchondral bone changes such as osteophyte formation [10, 23, 24]. Noninvasive medical imaging which is widely used for phenotyping OA based on these structural changes has proven to perform well in detection of early OA. However, the manual segmentation approach that is most commonly used is relatively time consuming and suffers from high observer variability especially in quantifying biomarkers such as cartilage thickness or volume. It is also inadequate to perform manual segmentation when the image data size is large [8].

Artificial intelligence or machine learning is an efficient approach to establish fully automatic algorithms that can identify early-onset OA biomarkers in numerous datasets in a standardized manner [24, 25]. Deep learning is a machine learning approach that is popular in the research field, and it has revolutionized computer-aided diagnosis using medical imaging as it has overcome the need of manual conventional techniques [26]. Studies over the past few years have shown that deep neural network architectures have been widely used in medical image analysis and have shown promising accuracy results in terms of classification, detection, and segmentation tasks in knee OA diagnosis. These tasks play a crucial role in early detection of OA [2, 8, 27]. Medical image analysis tools such as Imorphics (based in Manchester, UK), ArthroVision (based in Montreal, Canada), and Chondrometrics (based in Ainring, Germany) were developed to detect knee OA based on imaging biomarkers [8].

Different architectures of deep learning have been applied in different types of medical images from imaging modalities such as radiography, ultrasound, computed tomography, and MRI to diagnose knee OA. Among all the deep learning architectures, CNN architecture has gained a large amount of research interest, particularly in knee OA segmentations and diagnosis [26, 28, 29]. One of the main advantages of CNN is that they are easier to train and have fewer parameters compared to other architectures [30]. CNN, basically the U-Net architecture, is popularly used in knee OA for automated segmentation of the cartilage, menisci, bone, or total knee joint anatomy [31, 32]. Segmentation of the anatomical structures is important in the clinical practice to evaluate the disease progression and morphological changes where the recent breakthrough of this field is segmenting the cartilage from magnetic resonance (MR) images [28, 33].

In the study of OA pathophysiology, there are a variety of imaging modalities available in the healthcare and research sector where the choice depends on the specific role of the modality [28]. Previously, plain radiographs were the “gold standard” used for initial radiographic evaluation to diagnose or assess the severity of knee OA. The standardized knee radiography OA severity reading is Kellgren–Lawrence grading (KL-grade). Key pathological features of OA that can be easily obtained by radiographs are joint space narrowing (JSN) and formation of osteophytes [30, 34]. However, radiography not only exposes patients to radiation but also is incapable to characterize various structural phenotypes of knee OA, especially soft tissue structures such as cartilages, which are crucial for knee OA diagnosis [10].

Recent osteoarthritis research studies on fully automatic methods are mostly focused on MR images as they have excellent soft tissue contrast and distinct resolution on a knee joint. MRI is also a noninvasive technique that does not require ionizing radiation [8, 10, 35–37]. Although ultrasound imaging is a noninvasive, portable option that does not require ionizing radiation, its application is limited, especially on the segmentation tasks, due to the low contrast ratio and presence of speckle noise [38–40]. In addition, knee OA is a whole joint disease, which is a 3D complexity. Therefore, a 3D image of MRI can reflect the 3D structure of the knee joint while discriminating multiple tissue types and hence will provide a better interpretation of OA condition with a more detailed structure of the knee than two-dimensional (2D) radiography images [14, 16, 34]. MRI is not only capable for visualizing OA biomarkers as joint tissues, cartilage, and menisci, but it can also provide quantitative analysis of biomarkers such as cartilage volume, thickness, and biochemical changes depending on the MRI sequences [3, 6].

There are different types of MR imaging sequences such as dual energy steady state (DESS), turbo spin-echo (TSE), fast spin-echo (FSE), fast low angle shot (FLASH), spoiled-gradient echo (SPGR), gradient recalled echo (GRE), spin-echo spectral attenuated inversion recovery (SPAIR), and T1-weighted imaging sequence with fat suppression (FS) or water excitation (WE) [8]. To standardize knee MRI reading, scores such as Whole-Organ Magnetic Resonance Imaging Score (WORMS) or MRI Osteoarthritis Knee Score (MOAKS) have been developed [19].

## 4. Gap of Knowledge

There exist multiple reviews that have included deep learning in OA diagnosis. A number of OA review studies on knee joint segmentation have been published [8, 28, 41]. To the best of the authors' knowledge, no review article has addressed DL methods, specifically CNN on both segmentation and classification models, especially in 3D. The database used to find relevant articles was Web of Science. The keywords used were “Knee Osteoarthritis” AND (“Deep Learning” OR “Convolutional Neural Network”) AND “3D.” The search resulted in 107 articles. We excluded articles with irrelevant titles or abstracts to our study. A full-text reading was conducted to ensure all articles fall under the scope of this review. 43 articles from the search were excluded. 10 additional relevant works from the included article's reference list were included. There are a total of 74 studies reviewed in this paper. Review papers obtained from the search are summarized in Table 2.

**Figure 2:**
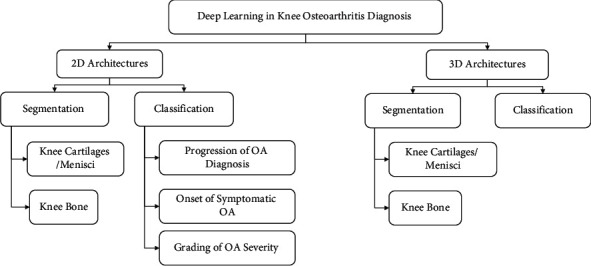
Taxonomy of the review.

The gap of the existing reviews [8, 10, 13, 24, 28, 41–45] is that none of the reviews highlighted 3D CNN as well as its importance in OA studies. In addition, most of the review papers focused on knee anatomical segmentation approaches instead of classification approaches in OA diagnosis. This review is focused on the evolution from 2D DL models towards 3D DL models, particularly CNN in OA diagnosis. As the adaptation of CNN is increasing in OA diagnosis, only CNN architectures will be covered in this review. This work presents all types of state-of-the-art CNN architecture and approaches in OA diagnosis, which also covers the highlights and improvement of the studies.  Figure 2 illustrates the taxonomy of CNN approaches in this review.

The rest of this review is organized into four sections. Sections 5 and 6 review the existing 2D and 3D architectures, respectively, for both segmentation and classification approaches. Based on the existing reviews, Section 7 discusses the performance of 3D CNN and its potential in the future direction of OA diagnosis.  Section 8 presents the conclusion of this review.

## 5. Application of 2D Deep Learning in Knee Osteoarthritis Assessment

### 5.1. Segmentation of Knee Cartilages/Menisci

Most of the techniques used in deep learning studies in the OA field are based on convolutional neural networks. A summary of 2D CNN-based segmentation approaches in the OA field is given in Table 3.

Majority of the CNN segmentation studies are based on the U-Net architecture, a symmetrical network consisting of the encoder and decoder, which first learns to encode by convolution downsampling and then decode into a segmentation mask which represents the object of interest in the image by upsampling the “deconvolutions” [20, 33]. There are several statistical evaluation metrics to justify a segmentation model's performance such as Jaccard similarity coefficient (JSC), Dice similarity coefficient (DSC), and Matthew's correlation coefficient (MCC). Among the metrics, DSC is commonly used to evaluate the similarity between the model's prediction output image against manual annotations or ground truth pixel by pixel [8].

Kompella et al. [37] adopted the state-of-the-art Mask R-CNN (regional convolutional neural network) for automated femoral cartilage (FC) segmentation from ultrasound 2D image scans. The ResNet-50 with the feature pyramid network was chosen as the backbone of the architecture with region proposal network to extract the region of interest (ROI). FC is then classified from ROI using the SoftMax classifier and results in a binary mask using the feature pyramid network. Preprocessed images with Gaussian filtering show better results, and networks pretrained with the COCO 2016 image dataset perform better than networks pretrained with ImageNet.

Several studies adopted the 2D encoder-decoder U-Net model proposed by Ronneberger et al. [52] in knee compartment segmentation studies. Norman et al. [46] utilizes 2D U-Net to segment six subcompartments of the knee, particularly articular cartilages and meniscus. Subjects with and without OA are both included in this study. Strong DSC has been reported in this model, especially on the 3D-DESS image dataset which is ranged between 0.753 and 0.878 for all subcompartments. The automated cartilage segmentation model has an efficient computational speed of averaged 5 seconds.

Similar to Norman et al. [46], Si et al. [47] decided to use 2D U-Net to segment the bones and articular cartilages of the knee from MR images which are femur, tibia, patellar, and each of their corresponding cartilages. The cartilages are segmented to obtain the cartilage thickness in 14 anatomical regions. The DSCs of cartilage compartments obtained from this study are in the range of 0.76–0.87. Wirth et al. [31] also used 2D U-Net for segmenting femorotibial cartilages to test the cartilage morphometry longitudinal test-retest reproducibility and had demonstrated high DSC for both coronal FLASH and sagittal DESS images. For both studies by Si et al. [47] and Wirth et al. [31], only subjects without OA were included.

To overcome the lack of computational needs such as memory and training time requirement needed for 3D CNNs for 3D volumes segmentation tasks, Prasoon et al. [48] proposed a voxel classification system on 3D MR images using triplanar 2D CNNs. The CNNs are done on three orthogonal 2D patches on *xy*, *yx*, and *zx* planes of 3D images, respectively. The 3 CNNs were then fused and fed into the SoftMax classifier to perform tibial cartilage segmentation.

**Figure 3:**
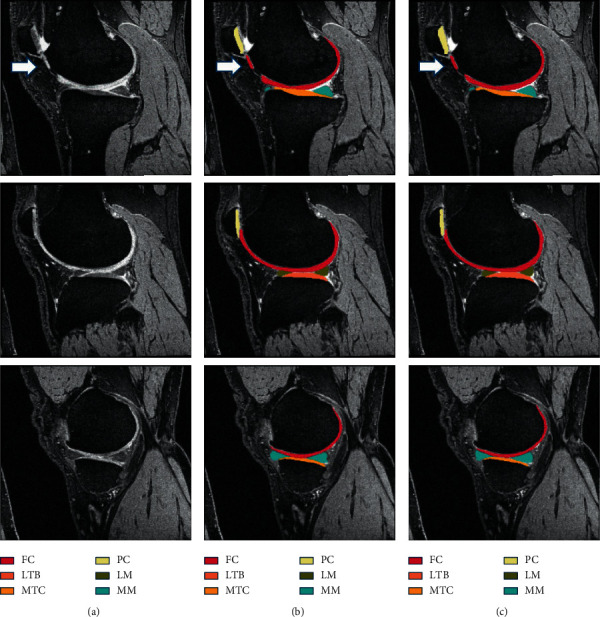
Examples of segmented MR images with different colour codes for different compartments (femoral cartilage (FC), lateral tibial cartilage (LTB), medial tibial cartilage (MTC), patellar cartilage (PC), lateral menisci (LM), and medial menisci (MM)) (adapted from [49]). (a) Original image, (b) manual segmentation, and (c) automatic segmentation.

There are several works that have made integrations or extensions to improve the existing models. Panfilov et al. [36] applied two regularization techniques, supervised mixup and unsupervised domain adaptation (UDA), to enhance the existing U-Net model on segmentation of articular cartilage and menisci. However, mixed results have been reported where mixup with weight decay potentially improves DSC performance, but UDA is relatively undesirable due to its heavy cost of computation. Combined UDA and mixup approach performed the worst. Byra et al. [32] demonstrated automatic menisci segmentation to assess quantitative evaluation on meniscus relaxometry. The model used is based on the U-Net architecture with transfer learning using 3D ultrashort echo time (UTE) cones sequences as input. Self-attention mechanism is utilized to enhance the segmentation performance. A high DSC of 0.860 and 0.833 is achieved. Given that performance of U-Net is limited by predefined loss functions, Gaj et al. [49] combined two deep learning networks to modify the objective function. The authors attempted the conditional generative adversarial networks (CGAN) model at integrating U-Net and had reported excellent cartilage and menisci segmentation performance on 3D-DESS images with a DSC in the range of 0.84–0.91.  Figure 3 shows the example of segmented MR images with different colour codes for different compartments including the articular cartilage and menisci.

### 5.2. Segmentation of the Knee Bone

**Figure 4:**
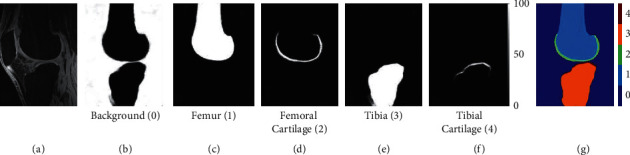
Example of knee bone and cartilage segmentation from pixelwise class probability with (a) original MR image, (b–f) background, femur, femoral cartilage, tibia, and tibial cartilage, with index 0 to 4, respectively, and (g) combination of each class index (adapted from [50]).

Liu et al. [50] presented automated segmentation of the knee bone and cartilage by combining 2D SegNet and 3D simplex deformable modelling. 3D deformable modelling allows desirable and smooth surface and shape of the final segmentation output. Results demonstrated that the 3D deformable modelling enhanced the segmentation accuracy of both models where the SegNet (Figure 4) outperforms the U-Net model.

Inspired by Liu et al. [50], Zhou et al. [51] extended the model by combining the 3D conditional random field (CRF) on 3D fast spin-echo (3D-FSE) MR image data sets. 3D CRF is integrated as a postprocessing step to ensure accurate labelling of the voxels. The multiclass tissue segmentation model reported DSC of more than 0.8 for most of the tissue types, especially the femur, tibia, and patella and their respective cartilages, as well as knee menisci.

### 5.3. Classification

The classification aspect of deep learning can be applied for predictive purposes in 3 main areas: progression of OA diagnosis, onset of symptomatic OA, and grading of OA severity.

#### 5.3.1. Progression of Osteoarthritis Diagnosis

A summary of 2D CNN-based classification approaches on progression of OA diagnosis is shown in Table 4. Among the deep learning classification works, most studies have compared deep learning model performance with other machine learning method's performance.

Schwartz et al. [12] have demonstrated that the CNN model is able to detect critical features and knee OA grade severity based on knee plain radiographs. This study classifies knee OA severity based on the International Knee Documentation Committee (IKDC) grading system. Results showed that when considering grades separately, CNN is achieving an intraclass correlation coefficient (ICC) of 0.685 with surgeons. Binary classification of IKDC D vs. other grading shows that the ICC achieved was 0.697, which is slightly higher. The study showed that CNN classifies knee OA as accurate as an arthroplasty surgeon.

Deep learning architectures have been proven feasible in detecting OA radiographic OA progression. Studies have presented that deep learning models using radiographic images outperformed the traditional model or clinical model that utilizes nonimaging data only [11, 53, 54]. Guan et al. [53] trained two CNN models which are VGG16 and DenseNet on knee radiographs to compare their knee OA prediction feasibility with support vector machine (SVM) on nonimage data. The clinical model based on SVM includes demographic and risk factors data to predict OA. The study showed that the combined SVM and deep learning model achieved the highest AUC of 0.832 followed by deep learning models with AUC more than 0.7 which is significantly higher than that of the clinical model. Next, Tiulpin et al. [54] developed CNN with the ImageNet pretrained model to perform multiclass classification to predict OA progression based on knee radiographs. The DL model is then compared to logistic regression which used demographic information of age, sex, BMI, and KL-grade as input variables. The DL model outperformed the logistic regression model with an AUC of 0.71. Guan et al. [11] developed an OA prediction model based on progression of medial joint space loss on knee radiography. The model proposed is the combination of two deep learning architectures for joint cropping and classification that are inspired from the You Only Look Once (YOLO) model and DenseNet, respectively. Similar to [53], the authors have attempted to integrate the DL model to extract features from knee radiograph and then joined them with demographic and radiographic risk factor data to form a joint training model. This approach resulted in a significantly higher AUC than of the traditional model that uses nonimage data, DL model, and joined DL and logistic regression model.

To address the lack of labelled training samples, Razmjoo et al. [7] presented semisupervised learning for OA progression prediction. Razmjoo et al. [7] proposed a predictive model using topological data analysis to construct graphs to feed into graph convolution network (GCN) as the input. Prediction of OA incidence is done by node labelling of different biomarkers and risk factors of the subjects simultaneously using graph-based analysis. The authors reported that the proposed semisupervised predictive model is a potential robust model.

Most studies are done on multiclass discrete classification on knee OA severity without conveying the actual continuous spectrum of OA progression. To overcome this issue, a Siamese neural network was developed by Li et al. [55] to perform binary classification of OA progression by assessing OA severity at single time points. The proposed model achieved an AUC of 0.9. The authors have reported that with image labelling, manual localization of ROI can be eliminated for the proposed method.

#### 5.3.2. Onset of Symptomatic Osteoarthritis

Table 5 displays the summary of 2D CNN-based classification approaches on prediction of onset of symptomatic OA.

Pierson et al. [56] trained a CNN to perform algorithmic pain prediction (ALG-P) which predicts pain based on knee X-ray. Similar to [11], the same authors, Guan et al. [22], combined YOLO and DenseNet to perform automated ROI cropping on knee radiographs and classification where it will be based on predicting pain progression. The DL model was then combined with risk factor data into a joint training model. The authors attempted similar performance comparison, and results demonstrated that the joint training model shows greater prediction performance than the deep learning and clinical model. Chang et al. [14] developed a Siamese network to classify knees with and without pain from 2D sagittal intermediate-weighted turbo spin-echo MR slices. The model achieved an AUC of 0.808 in assessing knee pain. The class activation mapping (CAM) saliency maps showed that effusion synovitis is present in most knee pain regions.

#### 5.3.3. Grading of Osteoarthritis Severity

A large proportion of the published study focused on utilizing deep learning to improve the grading of OA severity. A summary of 2D CNN-based classification approaches on grading of OA severity is presented in Table 6.

**Figure 5:**
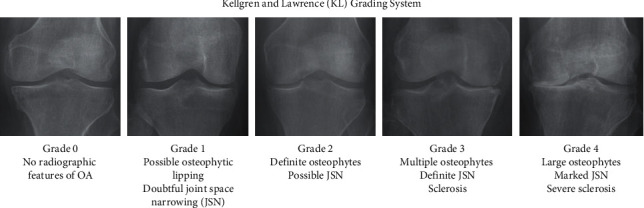
KL-grading system with corresponding knee joint samples (adapted from [34]).

Numerous studies utilize knee X-ray plain radiography in their classification model not only because it is commonly available and cost-efficient but also because the most significant hallmarks of OA are JSN and osteophyte formation which can be easily visualized by knee X-rays. Moreover, the JSN plays an important role in determining OA severity according to the KL-grade, which is a relatively commonly used grading by practitioners worldwide [16, 29, 59]. The KL-grading system (as shown in Figure 5) is categorized into five grades based on the ground truth where Grade 0 indicates no OA, Grade 1 indicates doubtful OA with minute osteophytes, Grade 2 indicates mild OA with definite osteophytes, Grade 3 indicates moderate OA with definite JSN and multiple osteophytes with possible bone deformation, and Grade 4 indicates severe OA where large osteophytes, JSN, severe sclerosis, and definite bone deformity are present [29, 34]. KL-grade defines OA severity as a composite score and is subjective based on practitioner's interpretation, hence causing a certain level of uncertainty in OA diagnosis [26].

To overcome this limitation, Tiulpin et al. [16] have developed a state-of-the-art approach using deep Siamese CNN to predict OA severity that presents probability distribution of the KL-grade and displays the highlighted OA features from knee radiographs by ensembling class discriminating attention maps. By highlighting the radiographic features, the model's decision-making process is made transparent which hence builds trust from the clinicians. The authors stated that the deep Siamese neural network allows the classification model to be more robust due to lower number of training parameters. The model achieved a high AUC score of 0.93.

Another study [5] was also interested in examining radiographic features identified for decision-making using saliency maps. Norman et al. [5] adopted U-Net in knee localization from radiographs where the localized images will be used to train the DenseNet neural network to classify OA severity, which is categorized into no OA, mild OA, moderate OA, and severe OA. DenseNet neural network utilizes dense blocks to allow feature learning from concatenating previous layers. Saliency maps have shown that osteophyte formation and joint space narrowing are the features identified by the network which are also biomarkers of OA. It was also found that the presence of hardware in the knee is one of the reasons of misclassification of OA severity by the algorithm.

Liu et al. [58] used Faster R-CNN, a deep learning approach that consists of a region proposal network (RPN) and Fast R-CNN to detect knee joints and perform classification based on KL-grading simultaneously. RPN plays a critical role in removing unwanted details from plain radiographs. Their proposed model involved novel loss function and larger anchors to enhance the performance of Faster R-CNN by addressing class imbalance and large input size issues. The proposed model performs better than the Faster R-CNN model. The authors acknowledge the limitations of this study where it is a supervised learning which requires a large amount of good quality annotated data to ensure classification accuracy and performance of the model.

Not only in the prediction of OA progression [7], semisupervised learning (SSL) has also been applied in staging OA severity and has demonstrated its advantage and effectiveness [23, 57]. To address the inadequacy of a large, annotated dataset, Nguyen et al. [57] extended the model from Tiulpin et al. [16] to perform a semisupervised learning method using the pi-model approach, where it adapted consistency regularization to ensure that the network behaves similarly on unannotated data. The proposed approach outperformed the approach in [16] and SL model in a limited data setting. The work in [16] was also extended in the study by Nguyen et al. [23] where the authors proposed a novel Semixup algorithm, which is also an SSL approach to automatically classify OA severity according to KL-grade based on knee radiographs. Semixup uses both in- and out-of-manifold regularizers with interpolated consistency for consistency regularization.

In another study, Zhang et al. [59] have proposed a state-of-the-art approach that has also shown better accuracy than that of approaches from [16]. Zhang et al. [59] applied different architectures of residual neural network (ResNet) to perform knee localization and prediction of KL-grade which were ResNet-18 and ResNet-34, respectively. ResNet-34 from [16] is modified and joined with the convolutional block attention module (CBAM) mechanism and suggested that CBAM contributes to achieving high accuracy by generating more concentration on radiographic feature relevant regions.

Most studies predict OA severity based on discrete grading such as KL-grade. However, the approach by Antony et al. [30] has allowed the predictions to fall between grades, which correlate to the OA progression. Antony et al. [30] demonstrated different CNN model and regression loss in assessing knee OA severity based on mean squared area instead of binary or multiclass classification. This is because the authors proposed that the measure of OA severity is a continuous evaluation and hence, it is inappropriate to categorize OA in a discrete way. Comparisons were done between VGG16, VGG-M-128, and BVLC CaffeNet and trained linear SVMs. The findings suggested that fine tuning networks with regression loss have shown better classification performance.

Leung et al. [60] proposed a multitask DL model to diagnose OA severity based on KL-grade and predict the TKR possibility within 9 years using baseline plain radiographs. The model is based on the ResNet-34 architecture. The proposed model was compared with the binary outcome model based on KL-grade and OARSI grade and single-task learning DL model. The proposed multitask model achieved the best performance compared to the single-task learning model and binary outcome models with an AUC of 0.87.

Unlike the KL-grade that works as a composite score, Osteoarthritis Research Society International (OARSI) allows grading of OA severity on different features independently. Tiulpin and Saarakkala [26] developed two deep residual networks incorporating squeeze-excitation (SE) and ResNeXt blocks to perform OA severity prediction from plain radiographs based on KL-grading as well as OARSI grading. The authors reported SE-ResNet-50 and SE-ResNet-50-32 × 4d as a whole as their final model, achieving an AUC of 0.98. Kim et al. [17] utilized the SE-ResNet algorithm to compare deep learning algorithm performance with image data solely and with both image and nonimage datasets. The average AUC for DL with both image and nonimage data is higher than that with sole image data, significantly for OA severity of KL-grade of 2 and below. Hence, by adding patient data, the model is significantly efficient to detect early OA.

**Figure 6:**
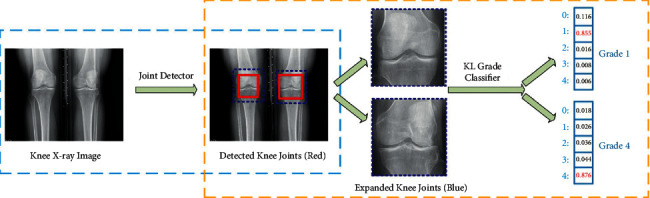
Example of knee joint severity grading staging, which includes knee joint detection and knee KL-grade classification (adapted from [34]).

Similar to the works mentioned earlier regarding the classification-based approaches on knee pain [11, 22], Chen et al. [34] also proposed two deep CNN approaches in knee OA diagnosis based on KL-grading (Figure 6). The YOLOv2 network was used to localize knee joints as an input for the classification networks, which are the variants of ResNet, DenseNet, InceptionV3, and VGG architectures. The fine-tuned models with the proposed ordinal loss perform better than models with cross-entropy loss. For this study, the VGG-19 with the proposed ordinal loss model achieved the best KL-grade classification performance.

Although knee radiographs have shown significant performance in OA diagnosis, it is unable to identify early OA as the best measure of early OA only can be detected through degeneration of articular cartilage, which is a critical progression towards formation of JSN [16]. Hence, recent studies also investigate prediction of severity grading based on MRI images. Besides morphological features, biochemical analyses such as T1p and T2 relaxation time values are potential biomarkers of OA as well [46, 61]. Voxel-based relaxometry has shown feasibility in both segmentation and classification studies. The relaxation time measurements help to predict the degradation levels of the collagen in the cartilage and menisci [25]. Pedoia et al. [61] proposed a deep learning approach, DenseNet, to classify the presence of OA by learning one feature from the MR images, which is the T2 relaxation times only, and included other demographic information.

Under the big umbrella of deep learning, besides CNN, various non-CNNs have been applied in segmentations and classifications in knee joints for OA studies. For example, conditional generative adversarial networks (CGANs) [62] have been applied in segmentation of multiple knee joint tissues whereas holistically nested network (HNN) has demonstrated bone segmentation [33]. However, non-CNNs such as dense neural network [63] and discriminative regularized auto-encoder (DRAE) [2] have been proposed in the classification task.

Meanwhile, Wahyuningrum et al. [29] proposed a deep learning approach that applies a CNN, VGG16, to extract features from plain radiography and a non-CNN, long short-term memory (LSTM), to classify knee OA severity based on KL-grading. LSTM is a type of recurrent neural network (RNN). The results showed that the proposed method achieves better accuracy than previous approaches [9, 16, 34] with a relatively shorter computing time.

## 6. Application of 3D Deep Learning in Knee Osteoarthritis Assessment

All the previous studies mentioned above utilized the 2D image or 2D slice from the 3D image as the model input to perform segmentation and classification. Several 2D CNN studies on OA proposed 3D CNN as a future work or recommendation to improve OA diagnosis. 3D networks require higher GPU memory and computation performance which can be accomplished with the technologies available today.

### 6.1. Segmentation of Knee Cartilages/Menisci

A summary of 3D CNN-based segmentation approaches in the OA field is presented in Table 7.

Marzorati et al. [64] performed automatic femur and tibia segmentation from CT images to extract pathological OA features. Results presented that implementation of 3D-U-Net in bone segmentation outperformed 2D-U-Net despite having more processing layers. Although CT provides excellent 3D visualization, recent 3D studies on OA diagnosis show limited attention to CT images and are more concentrated on 3D MR images. One of the main drawbacks is that CT exposes radiation to patients [28].

Various DL methods on knee cartilage segmentation using MR images have been proposed. The MR image sequences acquired in most 3D CNN OA applications are 3D-DESS MR volumes [1, 3, 18, 21, 65, 66, 68–70].

“*μ*- Net” is one of the first 3D fully developed CNNs for knee cartilage segmentation proposed by Raj et al. [3]. The network architecture is inspired by U-Net to develop a multiclass segmentation of cartilages and menisci. The DSC scores achieved for various classes are 0.785 and above. A 3D U-Net CNN was utilized by Chaudhari et al. [65] to perform automated femoral segmentation to compare results from the image with original resolution and super-resolution by Deep Resolve and tricubic interpolation. Results conclude that super-resolution produces a more accurate segmentation than naive interpolation.

To obtain quantitative meniscal measures, Tack et al. [66] presented a 3D knee menisci segmentation model based on the combination of 2D U-Net, 3D U-Net, and 3D statistical shape modelling (SSM). The approach involves concatenation of 2D segmented mask to a 3D mask from which SSM will remove unwanted segmentation regions. Results conclude that medial meniscus extrusion is a potential biomarker to predict incident OA. Ambellan et al. [67] implemented a method similar to [66] which involves a four-step approach with additional SSM postprocessing, but on segmentation of knee cartilages and bones. The DSC scores reported are 85.6%–89.9% and 98.5–98.6% for both tibia's and femur's cartilage and bone. Tack and Zachow [68] adapted 3D U-Net to segment medial and lateral tibial cartilages via supervised learning methods. The authors claimed that this approach outperformed other approaches in articular cartilage segmentation [3, 66, 67].

Not only 3D morphology but also quantitative assessment such as cartilage thickness [69] and T2 [6] values which require segmentation of relevant compartments are important for OA indication. Iriondo et al. [69] have developed automated segmentation and cartilage thickness measurement algorithms to perform OA trajectory analysis. The segmentation performance obtained a DSC score of 0.874 for meniscus and 0.850–0.890 for articular cartilages. The study has validated that knees with nonstable cartilage thickness change rate are likely to represent OA incidence.

**Figure 7:**
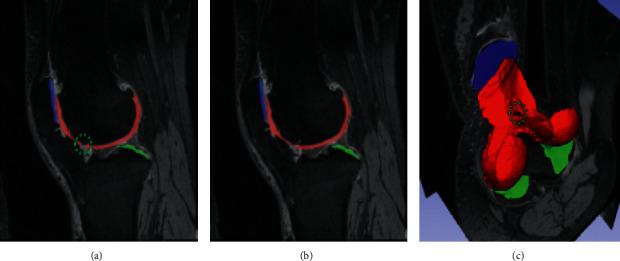
Example of 3D cartilage segment visualization results, with (a, b) sagittal views and (c) segmented cartilages (adapted from [70]).

Razmjoo et al. [6] utilized 3D V-Net to perform automated cartilage segmentation and quantification of T2 values. Five cartilage compartments were segmented with a DSC score of 0.57–0.75. Tan et al. [70] developed a 3D V-Net-like structured segmentation network with collaborative multiagent learning, where FC, TC, and PC are segmented separately and fused by the ROI-fusion layer.  Figure 7 shows the visualization results of 3D cartilage segmentation of the proposed method [70]. To address the lack of manual segmentation during training, Xu and Niethammer [27] proposed DeepAtlas, to jointly learn the weakly supervised image registration and semisupervised image segmentation. The segmentation model is customized from a light 3D U-Net design with smaller feature size to accommodate the limitation of GPU memory.

2.5D multiplanar CNN allows combining 3D spatial information from several orthogonal image planes [51]. Lee et al. [71] performed a state-of-the-art bone-cartilage segmentation to extract the cartilage by removing the bone masks from the bone-cartilage complex. 2.5D segmentation is done by averaging the multiple segmentation masks on different plans with majority voting. The BCD-Net proposed results with a DSC score of 98.1% and 83.8% for femoral and tibial cartilages.

### 6.2. Segmentation of the Knee Bone

The authors of [1, 18] demonstrated that bone shape can be used as a predictive OA biomarker. The femur, tibia, and patella are converted into 2D spherical maps separately and then fused to form a three-channel image as an input into the classification model. Martinez et al. [1] modified a 3D V-Net architecture to predict OA incidence based on bone shape only. ResNet is utilized in the classification model as it outperformed other architectures with lower training parameters. Martinez et al. [18] concluded that integrating demographic data with bone shape features improves OA prediction accuracy.

### 6.3. Classification

There are relatively limited studies on 3D classification tasks, and the tasks are mostly based on MR images. A summary of 3D CNN-based classification approaches in the OA field is displayed in Table 8.

Tolpadi et al. [21] introduced 3D DenseNet to perform OA severity prediction and TKR prediction from MRI images. This is the first study to apply 3D CNN to predict TKR, based on OA severity. This study demonstrated that 3D MR image input outperformed 2D radiography image input in the model. As MRI allows visualization of soft tissues, it contributes to a better pipeline performance, as the biomarkers of TKR identified are the medial patellar retinaculum, gastrocnemius tendon, and plantaris muscle, which is more complex than that of determining OA progression. The model's AUC improved significantly when nonimaging data are included in the classification pipeline.

Pedoia et al. [20] demonstrated detection of meniscus and cartilage lesions to classify OA severity using full CNN. Automated segmentation of menisci and cartilages were done using 2D U-Net and were fed into 3D CNN lesion severity classifiers. Unlike the study in [20] that utilizes 2D CNN, Nunes et al. [19] applied two 3D V-Net approaches to optimize 11 class segmentation tasks. The study in [19] has attempted a novel multitask OA lesion detection approach by combining 2 3D-CNN DL classification models to identify cartilage lesions (CLs) and bone marrow edema lesions (BMELs) simultaneously. The model outputs a 3 class WORMS model after integrating demographics into the model. Both the studies [19, 20] used high-resolution 3D fast spin-echo (FSE) CUBE sequence and showed optimal performance by including demographics.

Not only in the field of OA, 3D CNN has also been applied in detecting anterior cruciate ligament (ACL) injuries from MR images [72]. Moreover, ACL damage has been known to be a high-risk factor of knee OA. Zhang et al. [72] have demonstrated that 3D DenseNet outperformed VGG16 and ResNet due to its low complexity and excellent anti-overfitting performance.

## 7. Discussion

In the past few years, deep learning emerged as a popular field of research in academia, especially in the field of medical imaging. In this work, we provided updates on the application of various CNN approaches in segmentation and classification models. Numerous publications on CNN models in the OA research have been either used alone or combined with other strategies such as non-CNN or SSM. Integrated approaches have shown to achieve excellent results, showing that it is good to combine different techniques to improve the CNN performance.

From our review, it is obvious that most studies utilize plain radiography as their architecture input for classifying OA. However, changes in the knee joint are multidimensional; hence, a 3D image will provide a better representation of the joint changes than 2D images. In addition, one of the key indicators of OA progression involves delineation of articular cartilages (femoral cartilage, tibial cartilage, and patellar cartilage), which is not visible on plain radiography. The 3D morphology and qualitative assessment of the cartilages play an important role in the evaluation of OA [70].

Despite MRI being the ideal modality for OA assessment considering its excellent soft tissue contrast that enables advanced 3D biomarker cartilage visualization, there is a lack of MRI-based 3D CNN application for OA diagnosis [20, 21]. We observed that the publication on MRI-based classification models are limited as well, which might be due to the lack of recognition or feasibility of the MRI-based grading as compared to the KL-grade which is widely used in clinical practice. The MOAKS and WORMS scoring are based on multifeature of the MR images, which provide a more reliable evaluation of the whole knee joint. Hence, the use of 3D MRI approach in clinical practice and research should be encouraged. One of the reasons that this technique has not gained followers among the end users is the computational complexity.

Analysing the knee joint in a 3D view displays an accurate 3D structure of the joint, especially the articular cartilage, for a better visualization of OA diseases. 3D MR images alone might contain nonimaging data prediction such as pain experienced. It is also proven that 3D MR images result in a better pipeline performance than 2D radiographs, with higher sensitivity and specificity especially in early OA detection [21]. However, recent studies focused on 2D CNN architectures or adopting only subvolume of 3D images into 3D CNN as a compromise to the poor availability of computation size and limited GPU memory [28]. As 3D MR images are usually of high resolution and large size, simple adaptation of architectures might be inapplicable to 3D CNN [21]. Increasing complexity of the model might enhance the performance, but it will cause the training of the model to be computationally heavy and the cost of computation to be expensive. Although 3D CNN is more computationally challenging [64], 3D CNN models show similar accuracy to the experts in computer-aided diagnosis performance [68]. Hence, future research can investigate the optimization of 3D CNNs by reducing the architecture's complexity and the training parameters. There is also potential advancement of the performance, by training the model well with optimal learning parameters. In addition, recently, there is a memory-efficient solution for 3D image segmentation that has been proposed by Heinrich et al. [73] which may contribute to the emergence of more 3D CNN research studies [67].

## 8. Discussion

Artificial intelligence has shown excellent similarity with human experts in performance of detection and classification applications of OA diagnosis, especially deep learning where it intelligently learns the features directly from the raw data. It will be an open challenge on 3D CNN using MRI images in OA diagnosis. First, there are various types of 3D MRI sequences which have been used across different studies, which make direct comparisons of the approaches inapplicable as MRI sequence may affect the results obtained [20]. This makes the comparison between studies and their approaches difficult. Second, there is a lack of a large-scale ground truth [8]. Ground truths are manually annotated medical images by experts used to assess the performance of the computational model. A limited amount of ground truths might lead to overfitting of the model. A potential way to eliminate the need of well-segmented ground truth images by experts is to replace well-segmented ground truth images with region-based ground truth images. Region-based ground truth images are easier to obtain because splitting into subcropped images is simpler than performing pixelwise segmentation [74]. Finally, it is the lack of a standard database as different databases might affect the accuracy resulting from the model [28]. To overcome the problem, the availability of public databases such as OAI, which has been widely used, contains enormous datasets that are suitable for future studies to compare their models. However, to ensure general applicability of the model, it is encouraged to adopt images from independent datasets to be included in the testing dataset of any DL model [36].

## 9. Conclusions

This study reviews the evolution of deep learning from 2D to 3D as a promising tool for computed-aided diagnosis for the knee osteoarthritis disease. The conventional approach to diagnose osteoarthritis is by examining medical images visually where manual assessment makes it difficult to identify the slightest progression of early-onset osteoarthritis. This is where the role of artificial intelligence comes in. In conclusion, deep learning holds significant promise in the development of osteoarthritis clinical decision aid.

As revealed by the literature above, CNN in medical imaging research has advanced significantly in recent years and has shown great potential in OA diagnosis. With the expanding availability of advanced computational power and data availability, 3D deep learning may greatly enhance the early diagnosis of knee osteoarthritis. This is significant in osteoarthritis diagnosis since a three-dimensional image allows the assessment of the knee joint from different planes and offers precise information of the disease's modest progression. However, to develop a robust and generalized 3D CNN in diagnostic application is still a challenging task and remains an open research area, not only considering the accuracy of the model but also the computational efficiency. Even though the application of 3D CNN is still in a preliminary phase, we envisioned that the development of 3D CNN methods based on MR images will offer better understanding on the progression of the OA disease, especially on early detection of OA in the knee joint. The future of clinical practice may utilize 3D automated clinical applications to embrace new possibilities, not only to detect biomarkers but also to show excellent performance on par with clinical experts in early detection of OA.

## Figures and Tables

**Table 1:** Summary of studies conducted with nonimaging data.

Publication reference	Task	Data set (nonimaging data)	Performance	
			With nonimaging data	Without nonimaging data
Lim et al. [9]	Predict presence of OA	5749 subjects with 24 features including demographics, personal characteristics, lifestyle variables, and health status (3795 training (30% validation), 1955 testing)	AUC: 76.8%	—
			Accuracy: 71.97%	
			SN: 66.67%	
			SP: 73.35%	
			Positive predictive value: 39.53%	

Christodoulou et al. [4]	Predict progression of OA	OAI: 4796 subjects with 141 features including joint symptoms, disability, functionality, lifestyle, and general health status	Overall accuracy: 79.39%	—
Guan et al. [11]	Predicting progression of radiographic medial joint space loss	7 features of demographic data and radiographic risk factors	AUC: 0.863; SN: 80.5%; SP: 80.5%	AUC: 0.799; SN: 78.0%; SP: 75.5%
Kim et al. [17]	Predict knee OA severity based on KL-grade	6 features including demographics, alignment, and metabolic data	AUC: 0.97 (KL0), 0.85 (KL1), 0.75 (KL2), 0.86 (KL3), and 0.95 (KL4)	AUC: 0.91 (KL0), 0.80 (KL1), 0.69 (KL2), 0.86 (KL3), and 0.96 (KL4)
Martinez et al. [18]	Detect OA and predict future onset OA	3 features including demographic data	Detecting OA: SN: 81.03%; SP: 79.01%	Detecting OA: SN: 79.0%; SP: 77.1%
			Predicting onset: SN: 76.77%; SP: 62.5%	Predicting onset: SN: 76.8%; SP: 57.5%

Nunes et al. [19]	Stage severity of cartilage lesion	3 features including demographic data	Accuracy: 86.7%	Accuracy: 82.79%
Pedoia et al. [20]	Detect and stage severity of meniscus and patellofemoral cartilage lesions	2 features including demographic data	Accuracy: 80.74% (normal), 78.02% (mild-moderate), 75% (severe)	Accuracy: 87.55% (normal), 71.43% (mild-moderate), 66.7% (severe)
Tolpadi et al. [21]	Predict total knee replacement	27 features including demographic data, health status, disability, pain scores	AUC± SD: 0.890 ± 0.021 (X-ray), 0.834 ± 0.036 (MRI)	AUC ± SD: 0.848 ± 0.039 (X-ray), 0.886 ± 0.020 (MRI)
Guan et al. [22]	Predict knee pain	7 features including demographic and radiographic risk factors	AUC: 0.804; SN: 75.2%; SP: 76.2%	AUC: 0.753; SN: 65.77%; SP: 73.51%

*Note.* Task: osteoarthritis (OA); performance: magnetic resonance imaging (MRI), specificity (SP), sensitivity (SN), and area under receiver operating characteristics curve (AUC).

**Table 2:** Summary of existing reviews.

Publication reference	Year	Scope of review	Modality
Pedoia and Majumdar [24]	2018	Advances in assessment (image processing and deep learning techniques), quantitative imaging, multidimensional data analysis of knee and hip OA	MRI, X-ray (plain radiography)
Hayashiet al. [10]	2019	MRI techniques on knee OA assessment: development of new concept and techniques, hybrid imaging, artificial intelligence application	MRI
Chaudhari et al. [25]	2019	Existing development in OA diagnosis using magnetic resonance images: Morphological imaging, compositional imaging, rapid biomarker extraction, hardware improvements	MRI
Garwood et al. [13]	2020	AI application on knee pathologies: cartilages (osteoarthritis), ligaments, meniscus, tendons, musculoskeletal ultrasound, bone tumors, fractures	MRI, X-ray (plain radiography)
Kaur et al. [41]	2020	Knee cartilage and bone segmentation approaches: thresholding-based, partial differential equation-based, graph-based, atlas-based, model-based, machine learning-based (includes deep learning)	MRI, CT
Gan et al. [8]	2020	Knee bone and cartilage segmentation approaches: region-based; deformable model-based, atlas-based, graph-based, classical machine learning-based, deep learning-based	MRI
		Evaluation of computational models, brief discussion of classification models	
Ebrahimkhani et al. [28]	2020	Knee articular cartilage segmentation approaches: conventional methods, active contour models, active shape and active appearance models, graph-based, atlas-based, learning-based (5 publications on deep learning)	MRI
Eckstein et al. [42]	2021	Imaging studies on OA research between January 2019 and April 2020: models of early knee OA, structure modification in established OA, deep learning approaches in image analysis	MRI, X-ray (plain radiography)
Kijowski et al. [43]	2019	Imaging studies on OA research between April 1, 2018, and March 30, 2019: risk factors of OA, OA disease evaluation or treatment response, technical advances, and deep learning in OA imaging	MRI, ultrasound, X-ray (plain radiography), CT, positron emission tomography (PET), dual energy X-ray absorptiometry (DXA)
Nieminen et al. [44]	2018	Imaging studies on OA research between 1 April 2017 and 31 March 2018: cross-sectional studies, prediction, prognostic and progression studies of different modalities and deep learning	MRI, radiography, CT, ultrasound, nuclear medicine
Saini et al. [45]	2021	Knee OA severity classification: distinct feature quantification-based, and composite grading-based	X-ray (plain radiography)
		Knee segmentation approaches: manual, semiautomatic, automatic methods	

*Note.* Scope of review: osteoarthritis (OA); modality: magnetic resonance imaging (MRI) and computed tomography (CT).

**Table 3:** Summary of 2D CNN segmentation approaches.

Publication reference	Region of interest	Modality (imaging sequence)	Data set	Network architecture	Performance
Kompella et al. [37]	FC	Ultrasound	256 images (training : validation: 85% : 15%)	Mask R-CNN	DSC: 0.80 (FC)

Norman et al. [46]	FC, lateral TC, medial TC, PC, lateral menisci, medial menisci	MRI (T1-weighted, DESS)	OAI: 174 images (121 training, 37 validation, 16 testing)	U-Net	DSC (T1-weighted): 0.742 (FC, lateral TC, medial TC, PC), 0.767 (lateral menisci, medial menisci)
					DSC (DESS): 0.867 (FC, lateral TC, medial TC, PC), 0.833 (lateral menisci, medial menisci)

Si et al. [47]	FC, TC, PC	MRI (sagT1-weighted, sagT2-weighted, corPDW FS, transversal PDW FS)	Tongren Hospital: 47 subjects (27 training, 20 testing)	U-Net	DSC± SD: 0.87 ± 0.01 (FC), 0.82 ± 0.01(TC), and 0.76 ± 0.04 (PC)
Wirth et al. [31]	Medial FC, lateral FC, medial TC, lateral TC	MRI (corFLASH, sagDESS)	OAI: 92 subjects (50 training, 21 validation, 21 testing)	U-Net	DSC± SD (corFLASH): 0.92 ± 0.02 (medial TC), 0.88 ± 0.03 (medial FC), 0.92 ± 0.02 (lateral TC), 0.88 ± 0.02 (lateral FC)
					DSC± SD (sagDESS): 0.91 ± 0.02 (medial TC), 0.89 ± 0.03 (medial FC), 0.92 ± 0.02 (lateral TC), 0.90 ± 0.02 (lateral FC)

Prasoon et al. [48]	TC	MRI (turbo 3D-T1-weighted)	(25 training, 114 testing) images	Three 2D CNN	DSC: 0.8249 (TC); SN: 81.92% (TC); SP: 99.97% (TC)
Panfilov et al. [36]	FC, TC, PC, menisci	MRI (DESS)	OAI: 88 subjects	U-Net-mixup-unsupervised domain adaptation	DSC±SD: 0.907 ± 0.019 (FC), 0.897 ± 0.028 (TC), 0.871 ± 0.046 (PC), 0.863 ± 0.034 (menisci)
Byraet al. [32]	Menisci	MRI (3D UTE cones)	University of California	2D attention U-Net	DSC: 0.860 (menisci)
			San Diego Institutional Review Board: 61 subjects (36 training, 10 validation, 15 testing)		

Gajet al. [49]	FC, lateral TC, medial TC, PC, lateral menisci, medial menisci	MRI (3D-DESS)	OAI: 176 images (122 training, 36 validation, 18 testing)	U-Net-conditional generative adversarial networks	DSC± SD: 0.8972 ± 0.023 (FC), 0.9181 ± 0.013 (lateral TC), 0.8609 ± 0.038 (medial TC), 0.8417 ± 0.058 (PC), 0.8950 ± 0.023 (lateral menisci), 0.8738 ± 0.045 (medial menisci)
Liu et al. [50]	FC, TC, FB, TB	MRI (T1-weighted SPGR)	SKI10: (60 training, 40 testing) images	SegNet + 3D simplex deformable modelling	ASD ± SD: 0.56 ± 0.12 mm (FB), 0.50 ± 0.14 mm (TB)
					VOE = 28.4 (FC), 33.1(TC)

Zhou et al. [51]	FC, TC, PC, FB, TB, PB, menisci	MRI (3D-FSE)	60 images	SegNet + conditional random field + 3D simplex deformable model	DSC ± SD: 0.97 ± 0.01 (FB), 0.962 ± 0.015 (TB), 0.898 ± 0.033 (PB), 0.806 ± 0.062 (FC), 0.801 ± 0.052 (TC), 0.807 ± 0.101 (PC), 0.831 ± 0.031 (menisci)

*Note.* Region of interest: femoral cartilage (FC), tibial cartilage (TC), patellar cartilage (PC), femur bone (FB), tibia bone (TB), and patella bone (PB); modality (imaging sequence): magnetic resonance imaging (MRI); data set: Osteoarthritis Initiative (OAI); network architecture: convolutional neural network (CNN); performance: Dice similarity coefficient (DSC), specificity (SP), sensitivity (SN), average symmetric surface distance (ASD), and standard deviation (SD).

**Table 4:** Summary of 2DCNN classification approaches on progression of osteoarthritis diagnosis.

Publication reference	Target tasks	Modality (imaging sequence)	Data set	Network architecture	Performance
Guan et al. [53]	Predict OA progression	X-ray (plain radiography)	OAI: 600 subjects (450 training, 50 validation, 100 testing)	Vgg16 and DenseNet	Vgg16: AUC: 0.717; SN: 80.0%; SP: 56.1%
					DenseNet: AUC: 0.744; SN: 94.1%; SP: 48.0%

Tiulpin et al. [54]	Predict OA progression	X-ray (plain radiography)	OAI: 5139 images (training)	CNN	AUC: 0.71
			MOST: 2,491 images (testing)		

Guan et al. [11]	Predicting progression of radiographic medial joint space loss	X-ray (plain radiography)	OAI: (1400 training, 150 validation, 400 testing) images	YOLO + DenseNet	AUC: 0.799; SN: 78.0%; SP: 75.5%
Razmjoo et al. [7]	Predict OA incidence	MRI	OAI: 1805 subjects	Topological data analysis (TDA) + graph convolutional network (GCN model)	Accuracy (F1): 0.91; SN: 0.84; SP: 0.99
Li et al. [55]	Predict OA progression by assessing severity	X-ray (plain radiography)	MOST: 3021 subjects (training : validation : testing; 80 : 10 : 10%)	Siamese neural network	AUC: 0.90

*Note.* Modality (imaging sequence): magnetic resonance imaging (MRI); data set: Osteoarthritis Initiative (OAI) and Multicenter Osteoarthritis Study (MOST); network architecture: convolutional neural network (CNN); performance: specificity (SP), sensitivity (SN), and area under receiver operating characteristics curve (AUC).

**Table 5:** Summary of 2D CNN classification approaches on onset of symptomatic osteoarthritis.

Publication reference	Target tasks	Modality (imaging sequence)	Data set	Network architecture	Performance
Pierson et al. [56]	Predict knee pain	X-ray (plain radiography)	OAI: 4,172 subjects (2877 training, 1295 validation)	CNN	AUC: 0.69
Guan et al. [22]	Predict knee pain	X-ray (plain radiography)	OAI: 2000 subjects (1500 testing, 200 validation, 300 testing)	YOLO + DenseNet	AUC: 0.753; SN: 65.77; SP: 73.51
Chang et al. [14]	Predict knee pain	MRI (SAG-IW-TSE)	OAI: 1505 subjects (training : testing; 90% : 10%)	Siamese network	AUC: 0.808

*Note.* Modality (imaging sequence): magnetic resonance imaging (MRI); data set: Osteoarthritis Initiative (OAI); network architecture: convolutional neural network (CNN); performance: specificity (SP), sensitivity (SN), and area under receiver operating characteristics curve (AUC).

**Table 6:** Summary of 2D CNN classification approaches on grading of osteoarthritis severity.

Publication reference	Target tasks	Modality (imaging sequence)	Data set	Network architecture	Performance
Tiulpin et al. [16]	Predict knee OA severity based on KL-grade	X-ray (plain radiography)	MOST: 18376 images (training),	Deep Siamese convolutional neural network	AUC: 0.93
			OAI: 2957 images (validation), 5960 images (testing)		

Nguyen et al. [57]	Predict knee OA severity based on KL-grade	X-ray (plain radiography)	OAI: 39,902 images (training)	Deep Siamese convolutional neural network with pi-model approach	Cohen's Kappa coefficient (KC): 0.790
			MOST: 3,445 images (testing)		Balanced accuracy (BA): 0.527

Nguyen et al. [23]	Predict knee OA severity based on KL-grade	X-ray (plain radiography)	OAI: 39902 images (training/validating)	Semixup (Siamese network + novel deep semisupervised learning)	Balanced accuracy ± SD: 71 ± 0.8%
			MOST: 3445 images (testing)		

Liu et al. [58]	Predict knee OA severity based on KL-grade	X-ray (plain radiography)	2770 images	Faster R-CNN (region proposal network + Fast R–CNN) + focal loss	Accuracy: 82.5%; SN: 78.2%; SP: 94.8%
Antony et al. [30]	Predict knee OA severity based on KL-grade	X-ray (plain radiography)	OAI: 8892 images	VGG16, VGG-M-128, and BVLC	Mean squared error: 0.504 (CNN-Reg)
				CaffeNet	

Norman et al. [5]	Predict knee OA severity based on KL-grade	X-ray (plain radiography)	OAI: 39,593 images (25,873 training, 7779 validation, 5941 testing)	DenseNet	SN: 83.7 (no OA), 70.2 (mild OA), 68.9 (moderate OA), 86.0 (severe OA) %
					SP: 86.1 (no OA), 83.8 (mild OA), 97.1 (moderate OA), 99.1 (severe OA) %

Zhang et al. [59]	Predict knee OA severity based on KL-grade	X-ray (plain radiography)	OAI: (38232 training, 10986 testing, 5422 validation) images	ResNet with convolutional block attention module (CBAM)	Accuracy: 74.81%; mean squared error: 0.36; quadratic Kappa score: 0.88
Leung et al. [60]	Predict knee OA severity based on KL-grade and predict total knee replacement	X-ray (plain radiography)	OAI: 728 subjects	ResNet-34 (ResNet with 34 layers)	AUC: 0.87
Tiulpin and Saarakkala [26]	Predict knee OA severity	X-ray (plain radiography)	OAI: 19704 images (training); MOST: 11743 (testing)	SE-ResNet-50 + SE-ResNet-50-32 × 4d (SE-ResNet-50 with ResNeXt blocks)	AUC: 0.98
Kim et al. [17]	Predict knee OA severity based on KL-grade	X-ray (plain radiography)	4366 images (3464 training, 386 validation, 516 testing)	Six SE-ResNet	AUC: 0.97 (KL 0), 0.85 (KL1), 0.75 (KL2), 0.86 (KL3), 0.95 (KL4)
Chen et al. [34]	Predict knee OA severity based on KL-grade	X-ray (plain radiography)	OAI: 4130 images (training : validation : testing; 7 : 1 : 2)	VGG-19 + proposed ordinal loss	Accuracy: 70.4%; mean absolute error (MAE): 0.358
Pedoia et al. [61]	Predict presence of OA	MRI (T2 mapping acquisition)	OAI: 4384 subjects	DenseNet	AUC: 83.44%; SN: 76.99%; SP: 77.94%

*Note*. Modality (imaging sequence): magnetic resonance imaging (MRI); data set: Osteoarthritis Initiative (OAI); network architecture: convolutional neural network (CNN) and squeeze-and-excitation (SE); performance: specificity (SP), sensitivity (SN), area under receiver operating characteristics curve (AUC), and standard deviation (SD).

**Table 7:** Summary of 3D CNN segmentation approaches.

Publication reference	Region of interest	Modality (imaging sequence)	Data set	Network architecture	Performance
Marzorati et al. [64]	Distal FB, proximal TB	CT	200 images (160 training, 20 validation, 20 testing)	U-Net	DSC: 96% (FB, TB); SN: 96% (FB, TB)
Raj et al. [3]	FC, TC	MRI (3D-DESS)	SKI10: 100 images (80 training, 20 testing)	*μ*-Net	DSC: 0.849 (FC), 0.8565 (lateral TC), 0.8066 (medial TC), 0.7847 (PC)
			OAI: 176 images (140 training, 35 testing)		

Chaudhari et al. [65]	FC	MRI (3D-DESS)	OAI: 176 images (124 training, 35 validation, 17 testing)	U-Net	DSC± SD: 90.2 ± 1.7% (FC)
Tack et al. [66]	Menisci	MRI (2D-DESS)	OAI: 1240 subjects (5 datasets)	2D U-Net (SSM); 3D U-Net	DSC (baseline): 83.8% (medial menisci), 88.9% (lateral menisci)
Ambellan et al. [67]	FC, TC, FB, TB	MRI: SKI10 (T1, T2, GRE, SPGR FS), OAI (DESS)	SKI10: (60 training, 40 validation, 50 testing) subjects	2D U-Net (SSM); 3D U-Net (SSM)	Imorphics: DSC ± SD (baseline): 89.4 ± 2.41 (FC), 86.1 ± 5.33 (medial TC), 90.4 ± 2.42 (lateral TC)
			OAI (Imorphics, ZIB): 88 subjects, 507 subjects		ZIB: DSC ± SD: 89.9 ± 3.60 (FC), 85.6 ± 4.54 (TC); ASD ± SD: 98.6 ± 0.30 (FB), 98.5 ± 0.33 (TB)
					SKI10: ASD ± SD: 0.43 ± 0.13 mm (FB), 0.35 ± 0.07 mm (TB)

Tack and Zachow [68]	TC	MRI (DESS)	OAI (Chondrometrics, Imorphics): 1378 subjects, 88 subjects	U-Net	Chondrometrics: DSC ± SD (baseline): 82.85 ± 5.53 (medial TC), 86.11 ± 4.37 (lateral TC)
					Imorphics: DSC ± SD (baseline): 88.02 ± 4.62 (medial TC), 91.27 ± 2.33 (lateral TC)

Iriondo et al. [69]	FC, TC, PC, menisci	MRI (DESS)	OAI (Imorphics): 176 images (1/3 training, 2/3 validation)	CNN	DSC ± SD: 0.890 ± 0.023 (FC), 0.880 ± 0.036 (TC), 0.850 ± 0.068(PC), 0.874 ± 0.024 (menisci)
Razmjoo et al. [6]	PC, lateral TC, medial TC, medial FC, lateral FC	MRI (MSME spin‐echo sequence)	OAI: 3921 images (training : validation : test set: 65 : 25 : 10%)	3D V‐Net	DSC ± SD: 0.75 ± 0.11 (lateral TC), 0.69 ± 0.13 (lateral FC), 0.68 ± 0.12(medial TC), 0.69 ± 0.11(medial FC), 0.57 ± 0.17 (PC)
Tan et al. [70]	FC, TC, PC	MRI (3D-DESS)	OAI: 176 images (120 training, 26 validation, 30 testing)	V-Net with adversarial network	DSC ± SD: 0.900 ± 0.037 (FC), 0.889 ± 0.038 (TC), 0.880 ± 0.043 (PC), 0.893 ± 0.034 (FC, TC, PC)
Xu and Niethammer [27]	FC, TC, FB, TB	MRI	OAI: 507 images (200 training, 53 validation, 254 testing)	DeepAtlas	DSC ± SD: 97.70 ± 0.65 (FB, TB),81.19 ± 3.47 (FC, TC), 89.45 ± 1.91 (FB, TB, FC, TB)
Lee et al. [71]	FC, TC	MRI (T1-weighted SPGR)	SKI10: (60 training, 40 validation) images	BCD-Net	DSC ± SD: 97.3 ± 1.9 (FB), 84.4 ± 4.1 (TB), 98.1 ± 1.1(FC), 83.8 ± 5.3(TC)
Martinez et al. [1]	FB, TB, PB	MRI (3D-DESS)	OAI: 40 images (25 training, 5 validation, 10 testing)	3D V-Net	DSC: 97.15% (FB), 97.28% (TB), 95.99% (PB)
Martinez et al. [18]	FB, TB, PB	MRI (3D-DESS)	OAI: 40 images (25 training, 5 validation, and 10 testing)	CNN	DSC: 88.9%–95.2% (FB), 87.0%–95.8% (TB), 85.1%–92.2% (PC)

*Note.* Region of interest: femoral cartilage (FC), tibial cartilage (TC), patellar cartilage (PC), femur bone (FB), tibia bone (TB), and patella bone (PB); modality (imaging sequence): computed tomography (CT) and magnetic resonance imaging (MRI); data set: Osteoarthritis Initiative (OAI); network architecture: statistical shape modelling (SSM) and convolutional neural network (CNN); performance: Dice similarity coefficient (DSC), specificity (SP), sensitivity (SN), average symmetric surface distance (ASD), and standard deviation (SD).

**Table 8:** Summary of 3D CNN classification approaches.

Publication reference	Target tasks	Modality (imaging sequence)	Data set	Network architecture	Performance
Tolpadi et al. [21]	Predict total knee replacement	MRI (3D-DESS)	OAI: 4790 subjects (3114 training, 957 validation, 719 testing)	DenseNet-121	AUC ± SD: 0.886 ± 0.020
Pedoia et al. [20]	Detect and stage severity of meniscus and patellofemoral cartilage lesions	MRI (3D-FSE CUBE)	1478 images (training : validation : testing: 65 : 20 : 15%)	3D CNN	AUC ± SD: 0.89 (menisci), 0.88 (cartilage); SN: 89.81% (menisci), 80.0% (cartilage); SP: 81.98% (menisci), 80.27% (cartilage)
Nunes et al. [19]	Stage severity of cartilage lesion	MRI (3D-FSE CUBE)	1435 images (training : validation : testing: 65 : 20 : 15%)	3D CNN	Accuracy: 86.7%
Zhang et al. [72]	Detect anterior cruciate ligament lesion	MRI (PDW-SPAIR)	(285 training, 81 validation, 42 testing) images	3D DenseNet	AUC: 0.960; accuracy: 0.957; SN: 0.944; SP: 0.940

*Note.* Modality (imaging sequence): magnetic resonance imaging (MRI); data set: Osteoarthritis Initiative (OAI); network architecture: convolutional neural network (CNN); performance: specificity (SP), sensitivity (SN), area under receiver operating characteristics curve (AUC), and standard deviation (SD).

## Data Availability

All the data used in this study are available in the list of references.
